# 207-nm UV Light—A Promising Tool for Safe Low-Cost Reduction of Surgical Site Infections. II: *In-Vivo* Safety Studies

**DOI:** 10.1371/journal.pone.0138418

**Published:** 2016-06-08

**Authors:** Manuela Buonanno, Milda Stanislauskas, Brian Ponnaiya, Alan W. Bigelow, Gerhard Randers-Pehrson, Yanping Xu, Igor Shuryak, Lubomir Smilenov, David M. Owens, David J. Brenner

**Affiliations:** 1 Center for Radiological Research, Columbia University Medical Center, New York, NY, United States of America; 2 Department of Dermatology, Columbia University Medical Center, New York, NY, United States of America; 3 Department of Pathology & Cell Biology, Columbia University Medical Center, New York, NY, United States of America; Georgetown University, UNITED STATES

## Abstract

**Background:**

UVC light generated by conventional germicidal lamps is a well-established anti-microbial modality, effective against both bacteria and viruses. However, it is a human health hazard, being both carcinogenic and cataractogenic. Earlier studies showed that single-wavelength far-UVC light (207 nm) generated by excimer lamps kills bacteria without apparent harm to human skin tissue *in vitro*. The biophysical explanation is that, due to its extremely short range in biological material, 207 nm UV light cannot penetrate the human stratum corneum (the outer dead-cell skin layer, thickness 5–20 μm) nor even the cytoplasm of individual human cells. By contrast, 207 nm UV light can penetrate bacteria and viruses because these cells are physically much smaller.

**Aims:**

To test the biophysically-based hypothesis that 207 nm UV light is not cytotoxic to exposed mammalian skin *in vivo*.

**Methods:**

Hairless mice were exposed to a bactericidal UV fluence of 157 mJ/cm^2^ delivered by a filtered Kr-Br excimer lamp producing monoenergetic 207-nm UV light, or delivered by a conventional 254-nm UV germicidal lamp. Sham irradiations constituted the negative control. Eight relevant cellular and molecular damage endpoints including epidermal hyperplasia, pre-mutagenic UV-associated DNA lesions, skin inflammation, and normal cell proliferation and differentiation were evaluated in mice dorsal skin harvested 48 h after UV exposure.

**Results:**

While conventional germicidal UV (254 nm) exposure produced significant effects for all the studied skin damage endpoints, the same fluence of 207 nm UV light produced results that were not statistically distinguishable from the zero exposure controls.

**Conclusions:**

As predicted by biophysical considerations and in agreement with earlier *in vitro* studies, 207-nm light does not appear to be significantly cytotoxic to mouse skin. These results suggest that excimer-based far-UVC light could potentially be used for its anti-microbial properties, but without the associated hazards to skin of conventional germicidal UV lamps.

## Introduction

Conventional germicidal UV lamps, typically emitting a broad spectrum of UVC light, are very effective at killing both bacteria and viruses [1, 2]. A particular advantage of UVC-mediated bacterial killing is that it is essentially independent of acquired drug resistance [3, 4]. The downside to the more widespread use of germicidal UVC radiation in populated hospital (or other) environments, is that it is a human health hazard, being both carcinogenic and cataractogenic [5–7].

We have developed an approach for UV-based sterilization using single-wavelength UVC light to kill bacteria, but potentially without harming human cells or tissues [8]. It involves the use of far-UVC radiation generated by inexpensive filtered excimer lamps [9] that emit primarily a single UVC wavelength [10, 11]; in particular, our approach has used a krypton-bromine (Kr-Br) excimer lamp that produces high-intensity light at 207 nm [10]. Excimer lamps are practical, inexpensive, and appropriately intense [10, 12].

The mechanistic background is that far UVC light in the wavelength range of around 200 to 220 nm is strongly absorbed by essentially all proteins [13, 14], and so its ability to penetrate biological material is very limited. For example the intensity of 207-nm UV light is reduced by half in about 0.3 μm of tissue, compared with about 3 μm at 250 nm and much longer distances for longer UV wavelengths [15, 16]. The very short half value distance of 207 nm UV light in biological material means that, while it can penetrate bacteria and viruses that are typically smaller than 1 μm in size [17–19], it cannot penetrate the human stratum corneum (the outer dead-cell skin layer, thickness 5–20 μm), nor the ocular cornea (thickness ~500 μm), nor even the cytoplasm of individual human cells.

Together with the eye [20, 21], the skin is the organ most at risk from UV damage [7] and we have previously shown *in vitro* that UV light at 207 nm produces no significant biological damage in a human skin 3-D tissue model [8]. Here we extend the 207-nm safety studies *in vivo in* a hairless mouse skin model using a fluence at which 207-nm UV excimer light and 254-nm light from a germicidal lamp are both highly effective for inactivating methicillin-resistant *Staphylococcus aureus* (MRSA), as assessed in our earlier *in-vitro* bactericidal studies [8].

## Materials and Methods

### Hairless mouse skin

Six to eight week old male hairless mice (SKH1-Elite strain 477) were purchased from Charles River Laboratories (Stone Ridge, NY). Hairless mice have UV action spectra for histological, physical and visible changes similar to those of human skin [22, 23]; they develop squamous-cell carcinomas following exposure to 254 nm germicidal UV lamps [24] and undergo edema and erythema following UV exposures [25, 26]. From the perspective of the range of 207 nm UV light, the typical thickness of the SKH1 hairless mouse stratum corneum is 5–10 μm [27, 28], which thus represents a useful model for human skin that has typical stratum corneum thicknesses from about 5 to 20 μm [29].

### UV lamps

We used an excimer lamp based on a krypton-bromine (Kr-Br) gas mixture that emits principally at 207 nm. The lamp (High Current Electronics Institute, Tomsk, Russia) was air-cooled with a 6,000-mm^2^ exit window [9]. A custom bandpass filter (Omega Optical, Brattleboro, VT) was used to remove essentially all but the dominant 207-nm wavelength emission [8]. A UV spectrometer (Photon Control, BC, Canada) sensitive in the wavelength range from 200 nm to 360 nm was used to characterize the wavelength spectra emitted by the excimer lamp, and a deuterium lamp standard with a NIST-traceable spectral irradiance (Newport Corp, Stratford, CT) was used to calibrate the UV spectrometer. Studies were also carried out with a conventional mercury germicidal lamp (Sankyo Denki G15T8, Japan) with peak UV emission at 254 nm used as positive control. An Ushio fluence meter (UIT-250; Cypress, CA) was used to measure the fluence rate from both the Kr-Br excimer lamp and the mercury lamp.

### Mouse irradiations

Nine SKH1 hairless mice (6 to 8 weeks old) were divided into three groups of three mice each: one group was exposed to 207 nm light to a fluence of 157 mJ/cm^2^ delivered over an 7 h period by a filtered Kr-Br excimer lamp. As a positive control, a group of three mice was exposed to the same UV fluence (157 mJ/cm^2^ delivered over 7 h) from a conventional germicidal lamp (254 nm) whereas another group was sham irradiated to zero UV fluence and constituted the negative control.

At this 157 mJ/cm^2^ fluence, 207-nm UV excimer light and 254-nm light from a conventional germicidal lamp are both highly effective at inactivating MRSA (survival fraction 4 × 10^−5^ and 4 × 10^−6^, respectively, as assessed in our earlier *in-vitro* bactericidal studies [8]).

**Fig 1:**
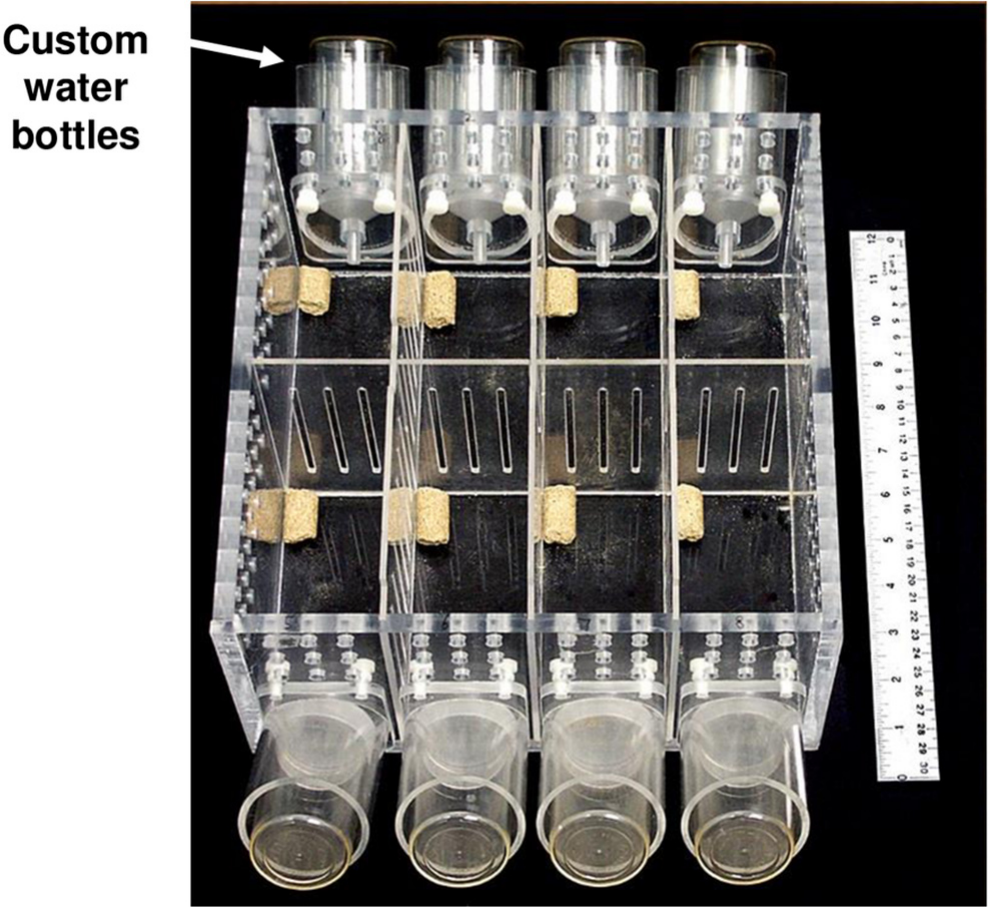
Top view of the custom-designed mouse UV irradiation box. The UV lamp is located above the irradiation box, which has separate compartments to house up to eight mice. A metal-mesh cover (not shown) allows UV light transmission (74% transparency) from above. As shown, during both acclimatization and irradiation, the mice have ad-libitum access to food and water.

Each mouse was housed unrestrained before (48 h acclimatization time) and during the UV exposures in one of the eight compartments of a specially-designed mouse irradiation box [60 mm (W), 125 mm (L) and 80 mm (H)] (Fig 1) covered with a metal-mesh top (74% transparency) to allow UV light transmission from above. Both before and during irradiation, the mice had ad-libitum access to water and Purina Laboratory Chow 5001 diet (St. Louis, MO). Mice were humanely sacrificed by asphyxiation 48 h after UV exposure after which the various assays were performed; all animal procedures were carried out in accordance with federal guidelines and protocols approved by the Columbia University Medical Center IACUC.

### Skin-Specific Endpoints

#### 1. Epidermal thickness and keratinocyte proliferation

At 48 h post-exposure, the mice were sacrificed and dorsal skin sections were stained with hematoxylin and eosin (H&E) for analysis of epidermal thickness. Briefly, excised dorsal mouse skin was fixed in 10% neutral buffered formalin overnight. Tissues were paraffin-embedded and cut in 6-μm sections. After subsequent tissue deparaffinization and rehydration, the sections were stained with Shandon Gill 3 hematoxylin and Shandon Eosin Y Alcoholic (Thermo Scientific, Somerset, NJ). For all the endpoints (unless otherwise noted), coverslips were mounted on the slides with Permount mounting medium (Fisher Scientific, Waltham, MA) and were examined using an Olympus IX70 microscope equipped with a Photometrics® PVCAM high-resolution, high-efficiency digital camera; Image-Pro Plus 6.0 software was used to analyze the images. For each mouse, the epidermal length was measured in nine randomly-selected fields of view.

An increase in epidermal thickness is often associated with an elevated expression of the cell proliferation marker Ki-67 antigen [30, 31]. Immunohistochemical analysis of Ki-67 was conducted as follows: Samples were deparaffinized and rehydrated and the DAKO Ancillary system K1499 (DAKO, Carpinteria, CA) was employed for antigen retrieval. Rehydrated tissue sections were immersed in target retrieval solution at 96°C for 20 min and then allowed to cool off for another 20 min. After three washes in 1X phosphate buffered saline (PBS), the specimens were blocked in protein blocking solution (1% BSA in PBS) for 1 h and then labeled at room temperature for 1 h in a humid chamber using 1:50 rabbit anti-Ki67 (Abcam, Cambridge, MA) in protein blocking solution. The sections were then washed and incubated with biotinylated secondary antibody at room temperature for 45 min and a rabbit-specific HRP/DAB (ABC) detection kit (Abcam, Cambridge, MA) was used for signal detection. For each mouse, the percentage of cells expressing Ki-67 was counted relative to the total number of cells [i.e. DAPI-stained nuclei with the coverslip mounting medium containing DAPI (Vectashield, Burlingame, CA)] in six randomly-selected fields of view (60x).

#### 2. Pre-mutagenic UV-associated DNA lesions

The immunohistochemistry protocol described above was used to assess UV-associated pre-mutagenic DNA lesions cyclobutane pyrimidine dimers (CPD) and pyrimidine-pyrimidone 6–4 photoproducts (6-4PP) [32, 33]. Specifically, skin tissues were incubated for 1 h in 1:1000 mouse anti-CPD or 1:300 mouse anti-6-4PP (Cosmo Bioscience USA, Carlsbad, CA) in 1% BSA in PBS. A mouse-specific HRP/DAB (ABC) detection kit (Abcam, Cambridge, MA) was used for signal detection. For each mouse, the percentage of cells showing dimers relative to the total number of cells (i.e. DAPI-stained nuclei) was counted in nine randomly-selected fields of view (60x).

#### 3. Skin tissue inflammation

Skin homeostasis and inflammation are regulated in part by dermal mast cells [34] that upon exposure to UV light increase in number with consequent excess production of inflammatory mediators [35–37]. To measure the number of mast cells in UVC-exposed dorsal skin, tissue slides were stained for 2 min with 10% toluidine blue dye in NaCl solution (Ricca Chemical, Arlington, TX) then washed and derehydrated.

In addition, UV triggers dermal infiltration of inflammatory cells such as neutrophils that exacerbate physical damage caused by UV light by releasing pro-inflammatory cytokines and reactive oxygen intermediates [38, 39]. We measured neutrophil infiltration through the expression of myeloperoxidase (MPO), the most abundant pro-inflammatory enzyme stored in these cells [40]. Briefly, samples were deparaffinized and rehydrated and antigen retrieval was conducted in the microwave using Tris-EDTA buffer (10 mM Tris base, 1 mM EDTA, 0.05% Tween 20, pH 9). Following washes in PBS, endogenous peroxidases were blocked by immersing the slides in 3.5% hydrogen peroxide for 20 minutes at room temperature. Slides were washed three times in PBS at room temperature and then incubated overnight at 4°Cwith rabbit anti-MPO (R&D Systems, Minneapolis, MN) at a concentration of 5 μg/mL in 1% BSA in PBS. After three washes in PBS, the slides were labeled with horseradish peroxidase conjugated goat anti-rabbit antibody (Jackson ImmunoResearch Laboratories, West Grove, PA) diluted 1:900 in 1% BSA in PBS for 1 h at room temperature. After rinsing the slides three times in PBS, color reaction was observed using a DAB peroxidase detection system (Sigma-Aldrich, St. Louis, MO) according to the manufacturer’s instructions. For each mouse, the number of mast cells / m^2^ and of MPO-expressing cells / m^2^ were counted in six randomly-selected fields of view (10x).

#### 4. Skin tissue differentiation

Together with cell proliferation and tissue inflammation, exposure to UV light can impact normal keratinocyte differentiation [41, 42], which is regulated by keratins. We focused on keratin 6A (K6A) that is markedly induced in stratified epithelia after UV exposure [43]. K6A expression was measured in skin tissues using the immunohistochemistry protocol described above; the antibody (BioLegend, Atlanta, GA) was diluted 1:3000 in 1% BSA in PBS and incubated at room temperature for 1 h. A DAB peroxidase system (Sigma-Aldrich, St. Louis, MO) was used for signal detection. Image analysis of keratin expression levels was performed with the Fiji / Image J software. For each mouse, the mean intensity of keratin expression was analyzed in nine randomly-selected fields of view (10x).

### Statistical Analysis

Comparisons of mean values between treatment groups and controls were performed using Student's t test, and comparison of proportions were assessed with standard χ^2^ tests.

## Results

### 1. Epidermal Thickness and Keratinocyte Proliferation

**Fig 2:**
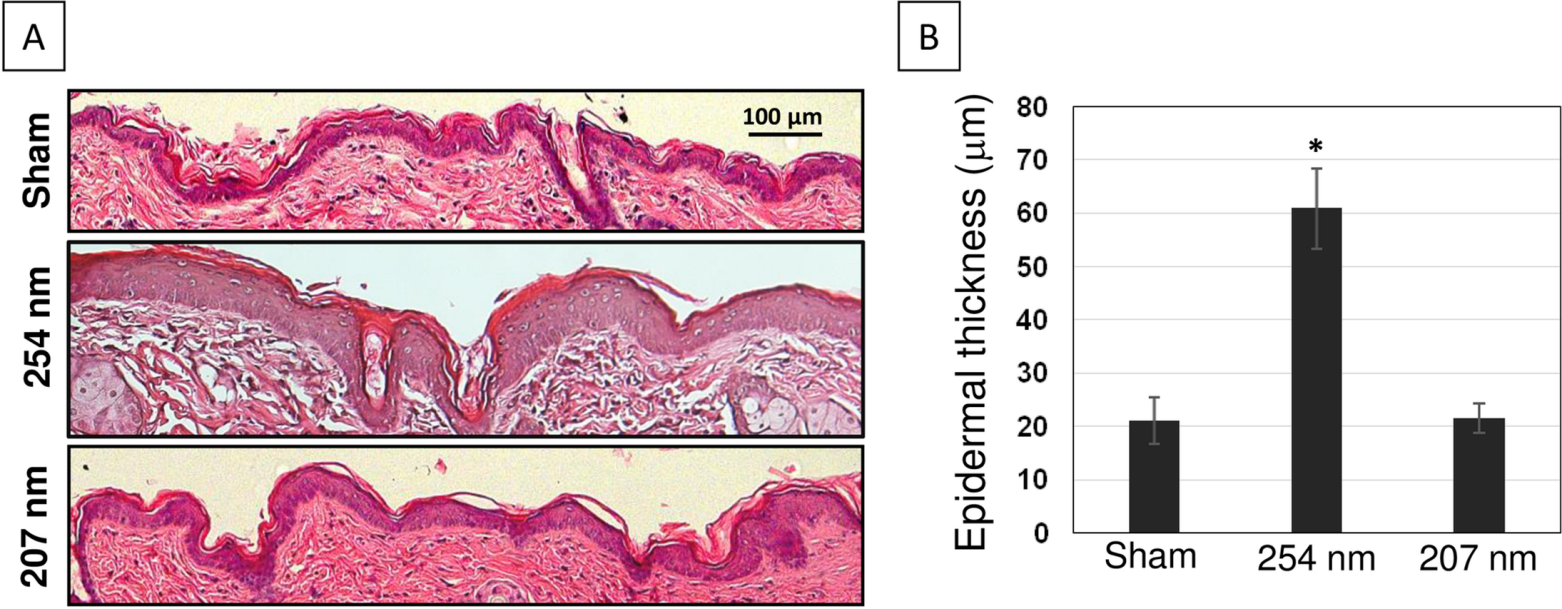
Epidermal thickness in hairless mice skin exposed to UVC light. A) Representative cross-sectional images of H&E-stained mouse dorsal skin comparing the epidermal thickness in sham-exposed mice (top panel), in mice exposed to 254-nm light (middle panel) or to 207-nm light (bottom panel). B) Quantification of epidermal thickness; values represent the average ± SD of epidermal thickness measured in nine randomly selected fields of view per mouse (n = 3).* p<0.0001.

At 48 h post-exposure, fixed dorsal skin sections were stained with hematoxylin and eosin (H&E) for analysis of epidermal thickness (Fig 2). Fig 2A shows typical H&E-stained cross-sections of dorsal skin of sham-exposed mice (top panel), of mice exposed to 254-nm light (middle panel) or to 207-nm light (bottom panel). Exposure to conventional germicidal UV light (254 nm) caused a ~ 2.8-fold increase in the average mouse epidermal thickness (p< 0.0001) (Fig 2B and Table 1). In contrast, the epidermal thickness of skin of mice exposed to the 207-nm excimer lamp was not statistically different from skin of unexposed mice (p = 0.54) (Fig 2B and Table 1).

**Table 1:** Summary of the combined results for each endpoint.

Endpoint	Controls	207 nm	207 nm p value	254 nm	254 nm p value
**Epidermal thickness (μm)**	21.8 ± 2.5	21.5 ± 2.8	0.54	60.9 ± 7.4	< 0.0001
**Ki-67 positive epidermal cells (%)**	18.2 ± 4.7	21.5 ± 5.4	0.19	36.1 ± 7.7	< 0.0001
**CPD positive keratinocytes (%)**	1.5 ± 0.5	2.2 ± 0.7	0.44	52.3 ± 5.6	< 0.0001
**6–4 PP positive keratinocytes (%)**	1.2 ± 0.3	1.4 ± 0.3	0.65	31.1 ± 1.4	< 0.0001
**Density of mast cells (/m**^**2**^**)**	45.0 ± 7.8	48.0 ± 1.3	0.42	86.6 ± 17.0	< 0.0001
**Density of MPO positive cells (/m**^**2**^**)**	35.0 ± 2.7	38.2 ± 1.7	0.76	202.4 ± 11.3	< 0.0001
**K6 positive keratinocytes (a.u.)**	15.7 ± 2.5	18.4 ± 3.8	0.14	45.8 ±6.9	< 0.0001

**Fig 3:**
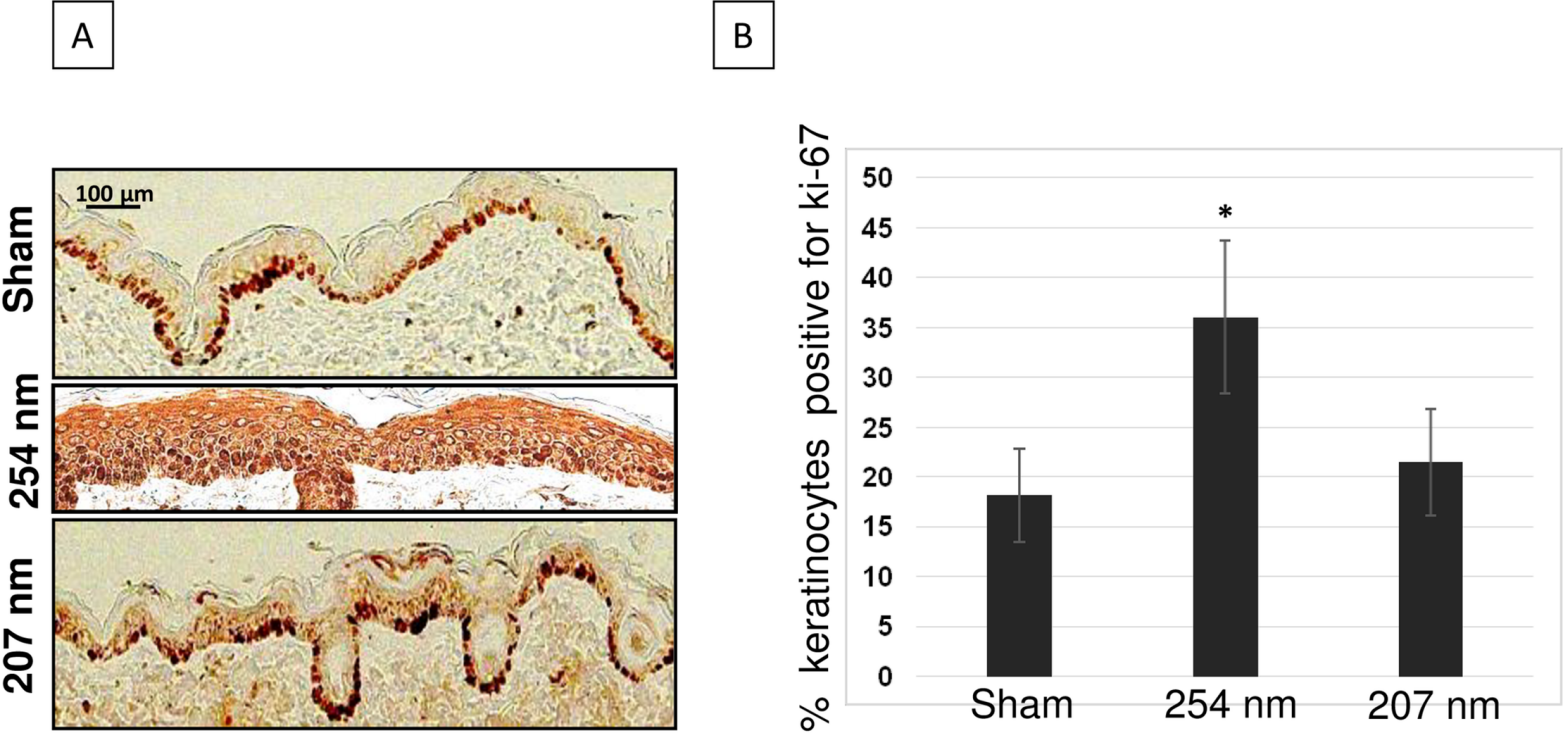
Expression of the proliferative marker Ki-67 in keratinocytes of hairless mice skin exposed to UVC. A) Ki-67-positive keratinocytes (dark-stained cells) in typical cross-sections of skin of sham-exposed mice (top panel), of mice exposed to 254-nm light (middle panel) or to 207-nm light (bottom panel). B) Quantification of the percentage of keratinocytes expressing Ki-67 antigen; values represent the average ± SD of Ki-67-positive cells measured in six randomly selected fields of view per mouse (n = 3). * p<0.0001.

254-nm light induced hyperplasia was associated to a hyper-proliferative epithelium as measured by the expression of the cell proliferation marker Ki-67 antigen. Fig 3A shows representative cross-sectional images of skin samples comparing Ki-67 expression (dark-stained cells) in sham-exposed mice (top panel), in skin exposed to 254-nm UV light (middle panel) or to 207-nm light (bottom panel). In skin of mice exposed to 254-nm UV light, the percentage of epidermal cells expressing the proliferative marker Ki-67 increased by 2 times (p< 0.0001) relative to sham-irradiated mouse skin (Fig 3B and Table 1). In contrast, Ki-67 expression in keratinocytes of skin exposed to the 207-nm excimer lamp was not statistically different from control (p = 0.19) (Fig 3B and Table 1).

### 2. Pre-Mutagenic UV-Associated DNA Lesions

**Fig 4:**
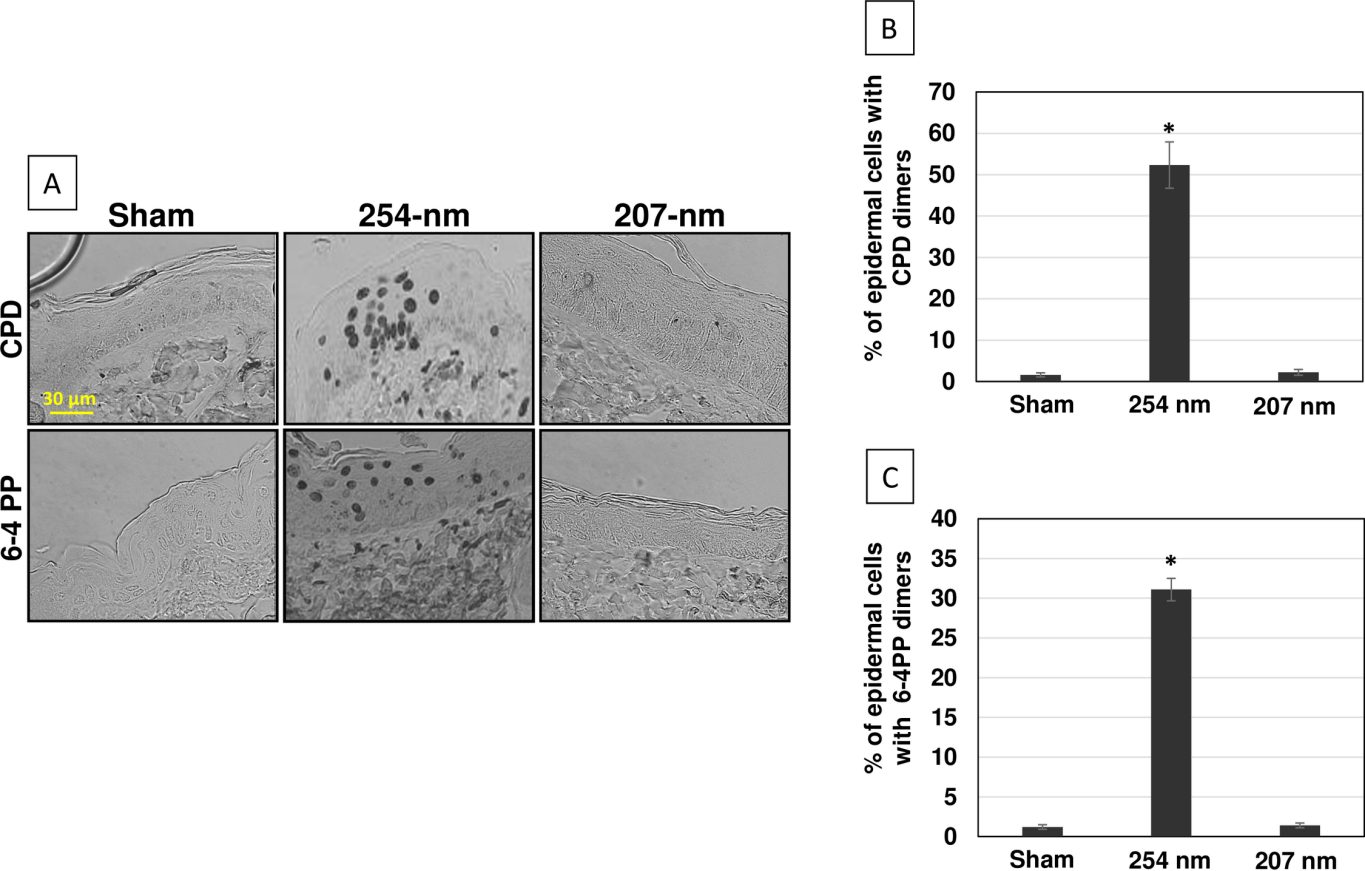
UVC-induced pre-mutagenic DNA lesions in hairless mice skin. A) Representative cross-sectional images of dorsal skin samples comparing pre-mutagenic skin lesions CPD (top row, dark-stained cells) and 6-4PP (bottom row, dark stained cells) in the epidermis of sham-exposed mice (left column), of mice exposed to 254-nm light (middle column) or to 207-nm light (right column). Quantification of the percentage of keratinocytes showing B) CPD or C) 6-4PP dimers; values represent the average ± SD of cells exhibiting dimers measured in nine randomly selected fields of view per mouse (n = 3). * p<0.0001.

Fig 4A shows representative cross-sectional images of skin samples from the three mice groups comparing pre-mutagenic skin lesions CPD and 6-4PP (dark-stained keratinocytes in the top and bottom row, respectively) with relative quantifications (Fig 4B and 4C, respectively). In agreement with our previous findings in a human skin model [8] and relative to controls, exposure to 254-nm light resulted in ~ 35-fold increase in CPD dimers and ~ 26-fold increase in 6-4PP dimers (p< 0.0001) (Fig 4B and 4C, and Table 1), whereas skin exposed to the same fluence of 207 nm UV light showed no such statistically significant increases (Fig 4B and 4C, and Table 1) for either CPD (p = 0.44) or 6-4PP (p = 0.65).

### 3. Skin Tissue Inflammation

**Fig 5:**
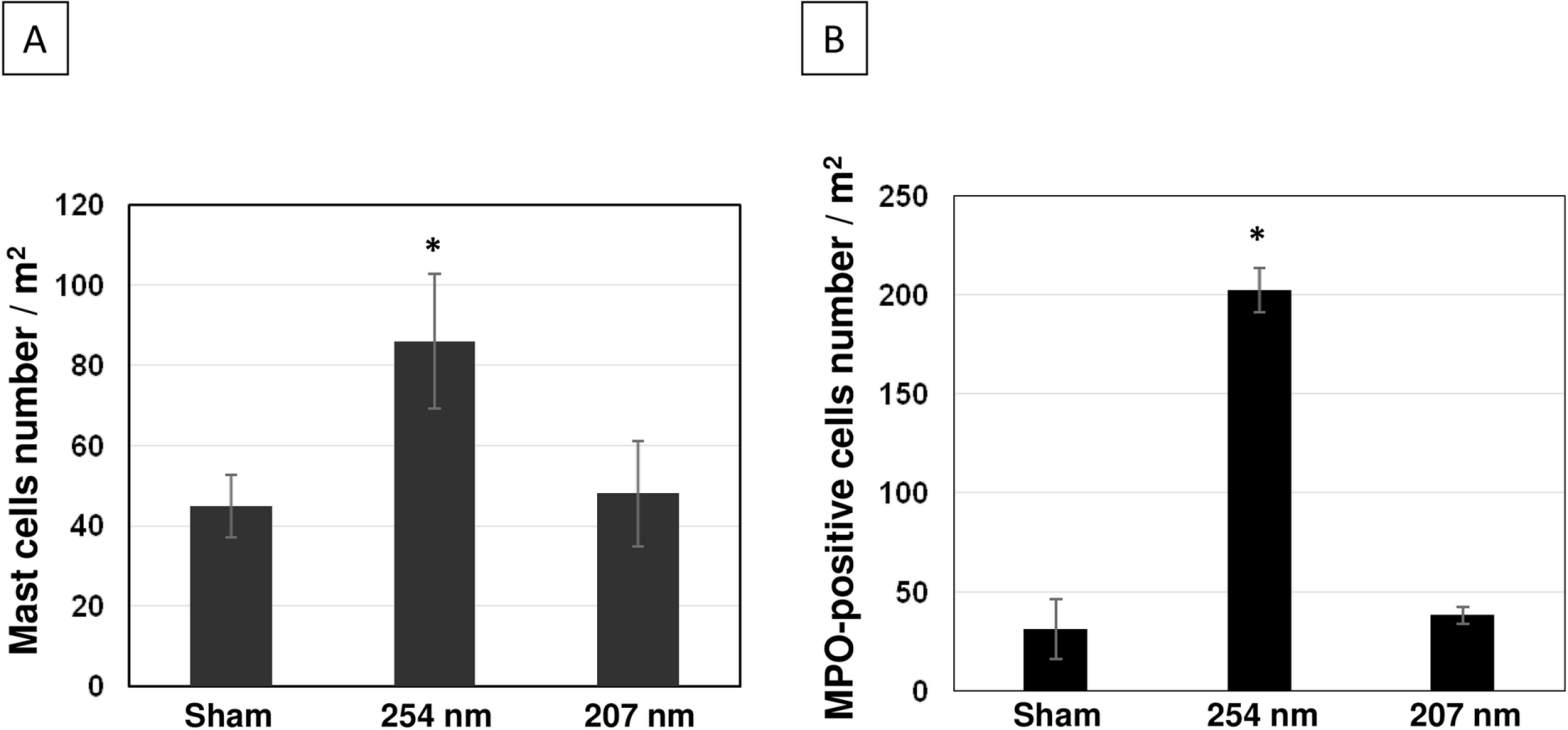
UVC-induced inflammation in hairless mice skin. Density of A) mast cells and B) cells expressing the myeloperoxidase (MPO) enzyme (i.e. neutrophils) in the epidermis of sham-exposed mice, of mice exposed to 254-nm light or to 207-nm light. Values represent the average ± SD of the number of cells / m^2^ measured in six randomly selected fields of view per mouse (n = 3). * p <0.0001.

To assess UV-induced tissue inflammation in dorsal skin samples of the three mice groups, we measured mast cell number by toluidine stain and the expression of the neutrophil myeloperoxidase (MPO) enzyme as a marker of neutrophil number [40]. Relative to control, mast cell density doubled in skin of mice chronically exposed to 254-nm light (p< 0.0001)(Fig 5A and Table 1), while neutrophil density increased by 5.8-fold (Fig 5B and Table 1). By contrast, in mouse skin chronically exposed to 207-nm light the density of mast cells (Fig 5A) and of cells expressing MPO (Fig 5B) was not statistically distinguishable from controls (p = 0.42 and p = 0.76, respectively).

### 4. Skin Tissue Differentiation

**Fig 6:**
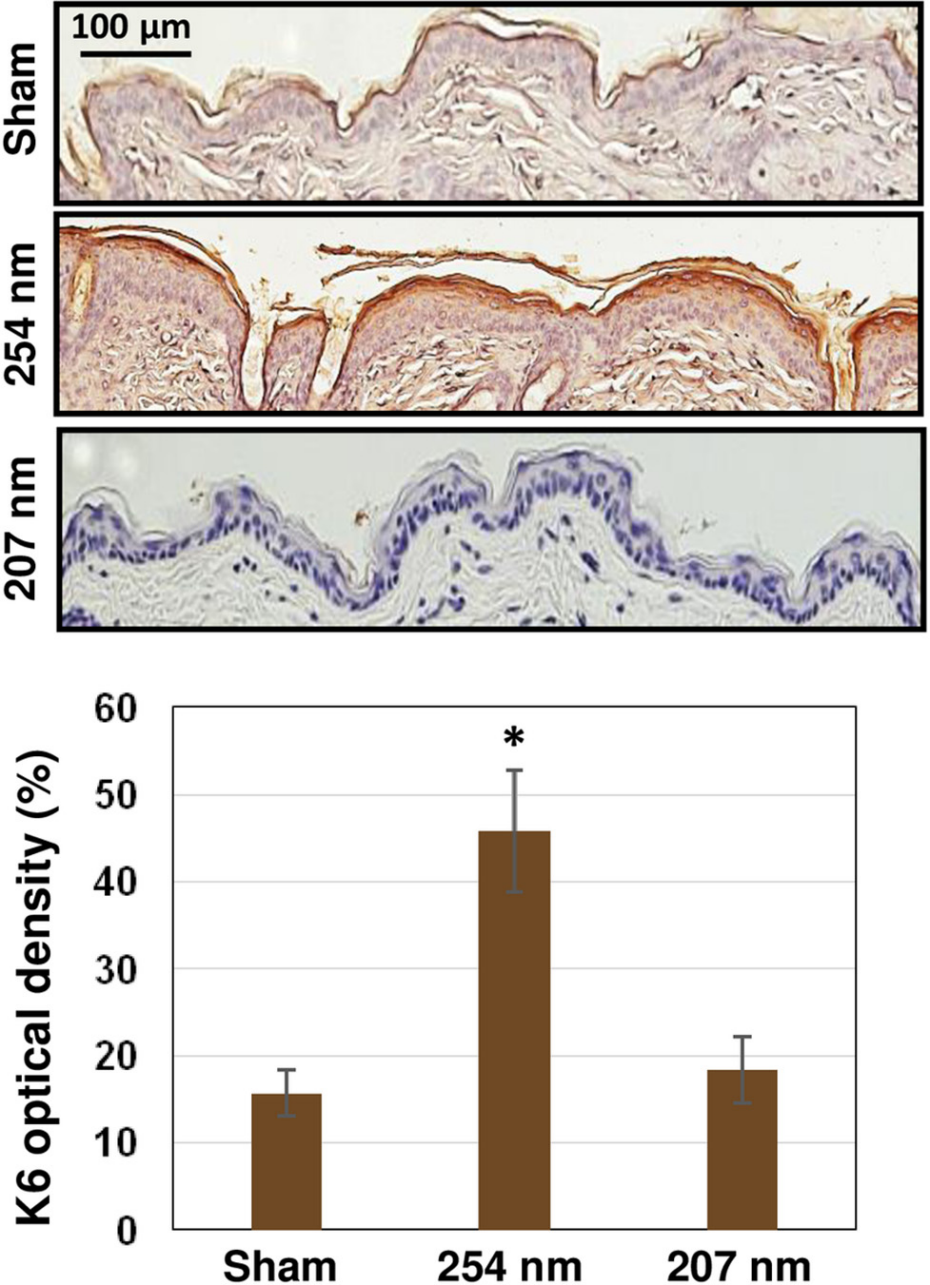
Tissue differentiation in UVC-exposed hairless mouse skin. **A)** Representative cross-sectional images of mouse dorsal epidermis expressing (brown stained area) K6A with B) relative quantification. Mice were sham-exposed (top panel), exposed to 254-nm light (middle panel) or to 207-nm light (bottom panel). Values represent the average ± SD of the percentage of keratin optical density measured in nine randomly selected fields of view per mouse (n = 3). * p <0.0001.

To examine skin keratinocyte differentiation in hairless mouse skin exposed to UVC light, we measured K6A expression. In agreement with previous findings [44, 45], newly synthesized K6A was increased by 3-fold in samples exposed to a conventional germicidal lamp (p< 0.0001) (Fig 6). In the case of skin exposed to 207-nm light, K6A expression level was not statistically different from that in unexposed skin (p = 0.14) (Fig 6 and Table 1).

## Discussion

In summary, we irradiated hairless mice skin *in vivo* with a germicidal fluence of 207 nm UV light, as well as with the same germicidal fluence of UVC light from a conventional germicidal lamp (254 nm), and compared both sets of results with controls from sham irradiations. We assessed a variety of endpoints relevant to UV-related skin damage; in all cases the 207 nm results were not statistically distinguishable from the results of control sham-irradiation, whereas the same UV fluence from a conventional germicidal lamp produced marked (and statistically significant) increase in response relative to control for all the damage endpoints studied.

These *in-vivo* mouse skin results are thus consistent with earlier studies in a 3-D human skin tissue *in vitro* model [8] that also showed significant skin damage after conventional 254-nm UVC irradiation, but none after 207-nm irradiation. The results are readily interpretable in terms of the limited range of 207 nm UV light that cannot penetrate the stratum corneum surface layer of mouse or human skin; by contrast, broad spectrum UVC from a 254 nm germicidal lamp can readily penetrate to the epidermis and dermis. We would expect that the conclusion drawn here would apply to all skin with a stratum corneum. Possible exceptions, therefore, might be non-keratinized regions of the oral mucosa that do not produce a stratum corneum, such as the inside of the cheeks, the floor of the mouth, and the underside of the tongue [46]- but these are likely to be of little relevance for our potential applications.

Together with our earlier studies suggesting that 207 nm UV light and 254 nm UV from a germicidal lamp are approximately equitoxic to MRSA bacteria [8], we may conclude that 207 nm light from an excimer lamp may have same antimicrobial properties as 254 nm UV light from a conventional germicidal lamp, but without associated skin damage risks.

This conclusion leads to potential applications such as reduction of surgical site infection (SSI), which continue to be a major issue in surgical environments [47–49]. Specifically, continuous conventional germicidal lamp irradiation of the surgical wound during surgery has been demonstrated to reduce SSI rates [50], but its use is not generally practical because of the associated skin cancer risks to the patient and staff. By contrast, if use of 207 nm UV excimer light can eliminate these health risks while maintaining anti-bacterial efficacy, continuous UV exposure of the wound during surgery becomes an attractive option for reducing SSI rates, including those due to drug resistant bacteria [3, 4]. The rationale for continuous excimer-light wound irradiation during surgery is that the majority of SSI result from bacteria alighting directly onto the surgical wound from the air [51–53]. Thus a continuous exposure of 207-nm UV light onto the surgical wound area during the surgical procedure might be anticipated to inactivate bacteria as they alight onto the wound area. Such a continuous exposure, as successfully used in earlier studies of surgical wound irradiation with conventional germicidal lamps [50], would be designed to inactivate bacteria and prevent the formation of bacterial clusters (biofilms) before they penetrated into the interior of the wound.

In conclusion, these studies provide *in-vivo* confirmation for earlier *in-vitro* based conclusions that 207 nm UV light at bactericidal fluences does not produce significant skin damage relative to controls. If it is indeed the case that excimer-based far-UVC light has similar antimicrobial properties compared with conventional germicidal lamps, but without the associated human health risks, the potential applications would be far reaching.
